# Supplementary material for: Magnetization reversal through an antiferromagnetic state
Source: Nat Commun. 2023 Aug 24;14:5174. doi: 10.1038/s41467-023-40722-y (PMC10449929; doi:10.1038/s41467-023-40722-y)
Supplement: Supplementary file 1 — Supplementary_Information [file 41467_2023_40722_MOESM1_ESM.pdf]

Supplementary Information for

Magnetization reversal through an antiferromagnetic state

S. Ghara,[1] E. Barts,[2] K. Vasin,[1,3] D. Kamenskyi,[1] L. Prodan,[1]
V. Tsurkan,[1,4] I. Kézsmárki,[1] M. Mostovoy,[2] and J. Deisenhofer[1]

[1] *Experimentalphysik V, Center for Electronic Correlations and Magnetism, Institute for Physics, University of Augsburg, D-86135 Augsburg, Germany*
[2] *Zernike Institute for Advanced Materials, University of Groningen, Nijenborgh 4, 9747 AG Groningen, The Netherlands*
[3] *Institute for Physics, Kazan (Volga region) Federal University, 420008 Kazan, Russia*
[4] *Institute of Applied Physics, MD-2028 Chişinău, Republic of Moldova*

(Dated: July 6, 2023)

Supplementary notes

1. Evaluation of polarization and magnetization

2. Evaluation of THz spectra and selection rule

3. Numerical simulations for calculating magnetization

4. Polarization calculations

Typeset by REVTEX

Supplementary note 1

Evaluation of polarization and magnetization:

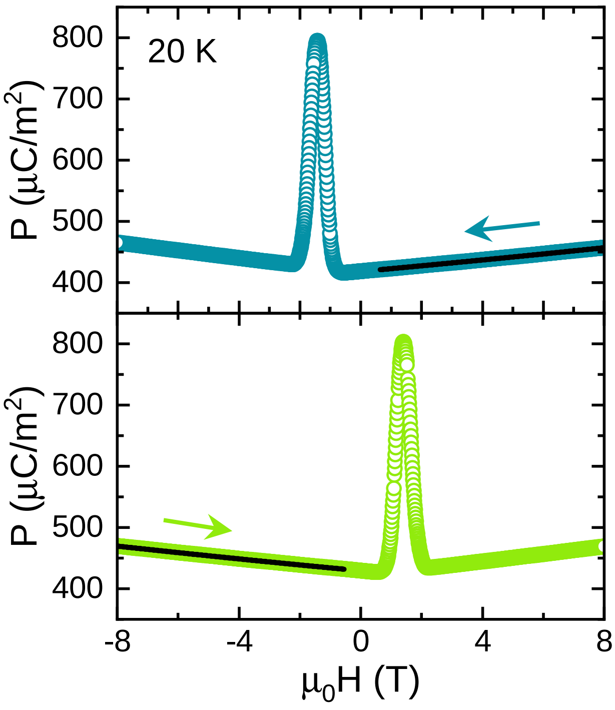

Supplementary figure 1: | **Linear ME effect in the FiM state.** Magnetic field-dependent electric polarization P at 20 K, where the magnetic field was varied from +8 T to −8 T (top) and from −8 T to +8 T (bottom). The solid black lines represent fits using $P = P_0 + \alpha H + \beta H^2$.

In Supplementary Fig. 1 we separately show the polarization curves in increasing and decreasing magnetic fields during the hysteretic cycle at 20 K and the corresponding fits of $P(H)$ using the fit function $P = P_0 + \alpha H + \beta H^2$. The values of P_0 and the linear (α) and quadratic (β) magnetoelectric coefficients were obtained by averaging the values from the up and down sweep and are given in Table I for the investigated temperatures. We found that the contribution of the

TABLE I: The values of P_0, linear (α) and quadratic (β) magnetoelectric coefficients in the ferrimagnetic state at various temperatures.

T (K)	P_0 ($\mu C/m^2$)	α (ps/m)	β (10^{-20}s/A)
13	397	6.0	8.3
15	397	5.5	7.4
20	424	5.8	6.8
25	420	7.3	−2.5
30	462	7.4	−22.5

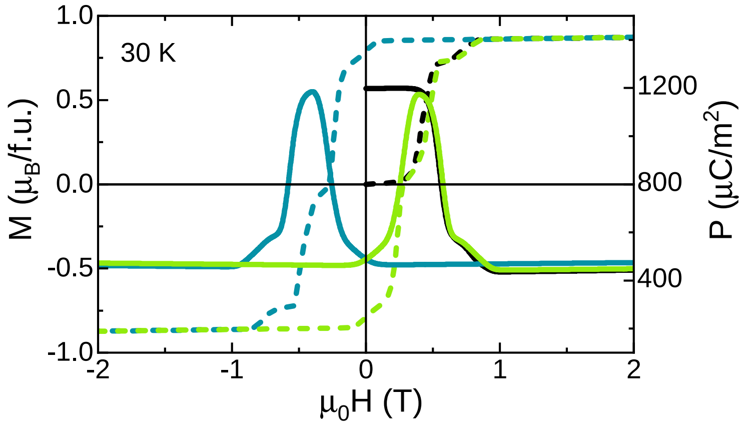

Supplementary figure 2: | **Magnetization and polarization at 30 K.** Magnetic field dependence of magnetization M (dashed lines) and polarization P (solid lines) measured along the c axis at 30 K.

quadratic magnetoelectric effect to the polarization is negligibly small, and thus, are not considered in calculating the antiferromagnetic volume fraction x_{afm}.

In Supplementary Fig. 2 we show the magnetic field-dependent magnetization and polarization at 30 K. Upon magnetization reversal, the polarization at the coercive fields reaches nearly the value of the pristine AFM state, which indicates that nearly the whole volume of the sample turns AFM at the coercive fields at 30 K. This is clearly shown in Supplementary Fig. 3, where we plot the magnetic field-dependent x_{afm} for three selected temperatures. This figure directly shows that, upon lowering temperature, the maximum value x_{afm}^{max} decreases, while the width of the symmetric distribution increases. In our numerical simulations, x_{afm}^{max} also decreases, whereas the width of the magnetization reversal region is approximately constant, as it is determined by

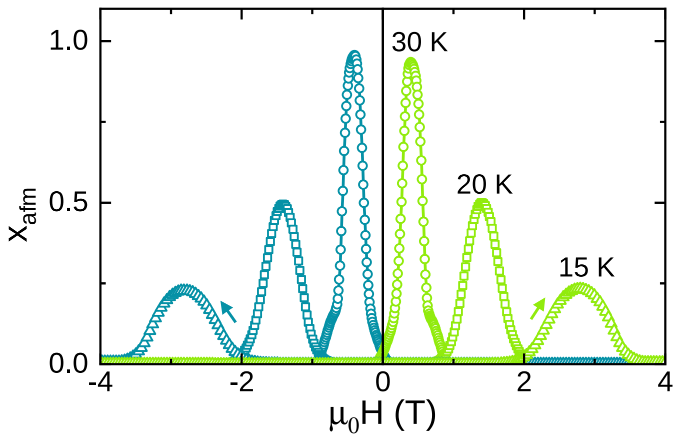

Supplementary figure 3: | **AFM volume fraction.** Magnetic field-dependent x_{afm}, obtained from the analysis of magnetization and polarization data, at three selected temperatures.

the number of Glauber spin-flip steps for a given magnetic field value, which for simplicity we keep temperature-independent.

Supplementary note 2

Evaluation of THz spectra and selection rule:

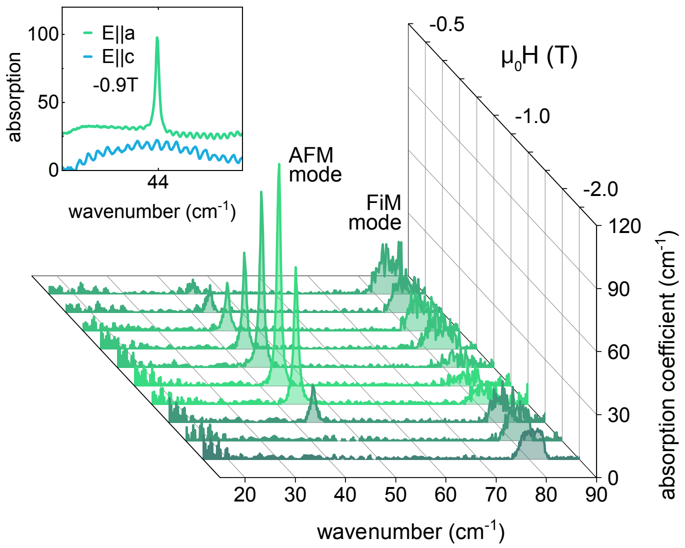

Supplementary figure 4: | **THz spectra at magnetization reversal.** Evolution of THz absorption spectra after subtraction of the underlying background in the field range of the magnetization reversal. The inset shows that the reemerging mode is electric-dipole active with polarization $E^{\omega} \parallel a$.

The reappearance of the characteristic AFM mode (its electric-dipole activity is shown in the inset of Supplementary Fig. 4) in the field range of the magnetization reversal is in perfect agreement with the analysis of the polarization in terms of the nucleation of the antiferromagnetic volume fraction x_{afm}. Consequently, we expect the integrated intensity of this mode to be proportional to x_{afm} and use

$$x_{\text{afm}} = \frac{\int_{\omega \in (\text{AFMmode})} \Delta A(\omega, H = H_c) d\omega}{\int_{\omega \in (\text{AFMmode})} \Delta A(\omega, H = 0) d\omega} \tag{S1}$$

to evaluate the ratio of the intensities of the AFM mode at the coercive field and in the pristine AFM state. The notation $A(\omega)$ stands for the absorption coefficient. The regions of the integration are indicated by the filled area of the AFM mode. The symbol ΔA denotes that we performed integration over the spectra with subtracted background (Supplementary Fig. 4). We approximated the background by smoothing each spectrum outside the frequency region of the AFM mode and

using a linear interpolation within this range. Analogously, we evaluated the intensity dependence of the ferrimagnetic mode using

$$x_{\text{fim}} = \frac{\int_{\omega\in(\text{FiMmode})} \Delta A(\omega, H = H_c) d\omega}{\int_{\omega\in(\text{FiMmode})} \Delta A(\omega, H = H_{sat}) d\omega} \tag{S2}$$

where the intensity of the FiM mode in the FiM state in magnetic fields larger than the saturation field H_{sat} was used as a reference for the volume fraction. By comparison of repeated measurements at 15 and 25 K, we estimated the standard deviation for the x_{afm} values derived for the AFM and the FiM mode to be about 1% and 15%, respectively. The larger experimental uncertainty for the FiM mode stems from its larger width and the increased signal-to-noise ratio of our experimental setup in the corresponding frequency range.

Note that this evaluation procedure is based on the fact that in linear response the absorption coefficient is assumed to be proportional to the density $n_V = N/V$ of N entities interacting with the radiation in the crystal of volume V. Hence, normalizing the absorption intensity in the metastable AFM state at the coercive field to the purely AFM state at $H = 0$ yields the corresponding ratio of the numbers of interacting centers $N_{afm}(H = H_C)/N_{afm}^{total}(H = 0)$. Whether this particular AFM absorption can be ascribed to A- or B-site iron ions or to a collective excitations of all magnetic ions is not clear at present, but one can safely assume that the interacting ions are homogeneously distributed in the corresponding volume fractions and hence we identify $x_{\text{afm}} = N_{afm}(H = H_C)/N_{afm}^{total}$. A similar argument holds for the FiM excitation. The remarkably good agreement with the volume fraction x_{afm} derived from the dc-values as shown in Fig. 3d justifies our assumptions.

Supplementary note 3

Numerical simulations for calculating magnetization:

Performing summation over projections of spins on A-sites, we can write the partition function of the model in Eq. 3 (given in main text) in the form

$$Z = \sum_{\sigma} e^{\beta M_B H \sum_{j\in B}\sigma_j} \prod_{i\in A} z_A(h_i), \tag{S3}$$

where $z_A(h_i) = \frac{\sinh\left(\frac{5}{4}\beta h_i\right)}{\sinh\left(\frac{1}{4}\beta h_i\right)}$ is the partition function of spins on the tetrahedral A-site i and h_i is an effective field on this site,

$$h_i = -\frac{\partial E}{\partial s_i} = -J_{\parallel} \sum_{j_{\parallel}(i)} \sigma_j + J_{\perp} \sum_{j_{\perp}(i)} \sigma_j + HM_A. \tag{S4}$$

Here, $j_{\parallel}(i)$ and $j_{\perp}(i)$ labels neighboring B-sites in the same ab layer and in neighboring layers, respectively. The product $\prod_{i\in A} z_A(h_i)$ in Eq.(S3) can be written as $e^{-\sum_{i\in A} u(h_i)}$, where $u(h_i) = -\frac{1}{\beta}\ln z_A(h_i)$ describes an induced temperature-dependent interaction between the Ising spins, as well as the interaction of the A-spin with the applied magnetic field. In particular, for $H = J_{\perp} = 0$, Eq.(S3) becomes a partition function of noninteracting layers of Ising spins on a triangular lattice with an effective nearest-neighbor interaction, $J_{\rm eff}(T)$:

$$e^{4\beta J_{\rm eff}(T)} = \frac{\sinh\left(\frac{15}{4}\beta h_i\right)\sinh\left(\frac{1}{4}\beta h_i\right)}{\sinh\left(\frac{5}{4}\beta h_i\right)\sinh\left(\frac{3}{4}\beta h_i\right)}. \tag{S5}$$

The critical temperature of the two-dimensional ordering of Ising spins, T_{2D}, is obtained from [1] as

$$e^{\frac{4J_{\rm eff}(T_{\rm 2D})}{k_{\rm B}T_{\rm 2D}}} = 3. \tag{S6}$$

For $T_{\rm 2D} = 60$ K (the ordering temperature of undoped $Fe_2Mo_3O_8$), one estimates $J_{\parallel} = 51.1$ K. More precise values of $J_{\parallel}$ and $J_{\perp}$ are obtained by fitting the experimental temperature dependence of the magnetization measured in different magnetic fields [2].

The average magnetic moment per f.u. is given by

$$M = M_B\frac{1}{N_B}\sum_{j\in B}\sigma_j + (1-x)M_A\frac{1}{N_A}\sum_{i\in A}B_2(\beta h_i), \tag{S7}$$

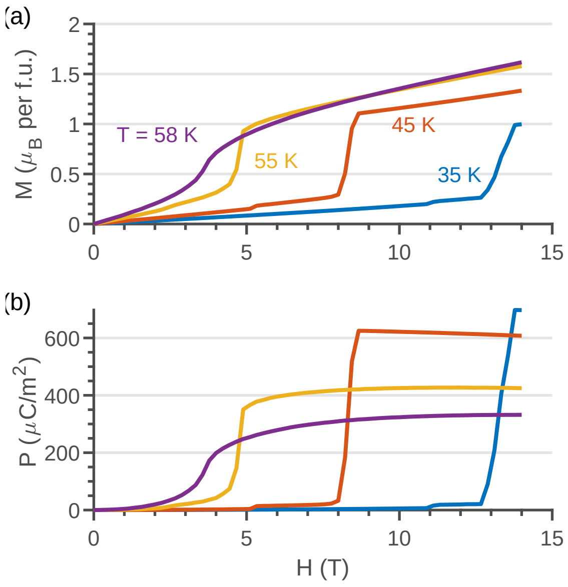

Supplementary figure 5: | **Numerically calculated $M(H)$ and $P(H)$ for pure $Fe_2Mo_3O_8$. a, b**, Field dependence of magnetization and polarization at different temperatures, respectively. The model parameters are $J_{\perp} = -1.2$ K and $J_{\parallel} = 47$ K.

where $N_A(N_B)$ is the number of A(B) sites, $x = 1 - \frac{N_A}{N_B}$ is the concentration of Zn dopants that substitute Fe ions on A-sites, and $B_2(x) = \frac{5}{4}\coth\frac{5x}{4} - \frac{1}{4}\coth\frac{x}{4}$ is the Brillouin function for $S = 2$. In particular, for the parent compound $Fe_2Mo_3O_8$,

$$M = M_B\langle\sigma\rangle + M_A\,\langle B_2(\beta h)\rangle\,. \tag{S8}$$

At low temperatures, $\beta h_i \to -\infty$, the strong antiferromagnetic intralayer exchange makes A-spins antiparallel to B-spins ($M_B > M_A$) in each ab layer. In this limit, the saturation magnetization of $Fe_2Mo_3O_8$ (per f.u.) is

$$M = M_B - M_A, \tag{S9}$$

whereas for Zn-doped $Fe_2Mo_3O_8$,

$$M = M_B - (1-x)M_A. \tag{S10}$$

At elevated temperatures, spins at nearly isotropic A-sites fluctuate more than the Ising spins at B-sites, which results in an increase of the average magnetic moment.

Supplementary Fig. 5a shows the field dependence of the magnetization M calculated using the Heisenberg-Ising model for undoped $Fe_2Mo_3O_8$ at four different temperatures and Supplementary Fig. 6a shows $M(T)$ under weak and strong magnetic fields. The calculated behavior of the magnetization is in good agreement with experimental data [2]. The temperature dependence of M for $Fe_{1.86}Zn_{0.14}Mo_3O_8$ is shown in Supplementary Fig. 7a.

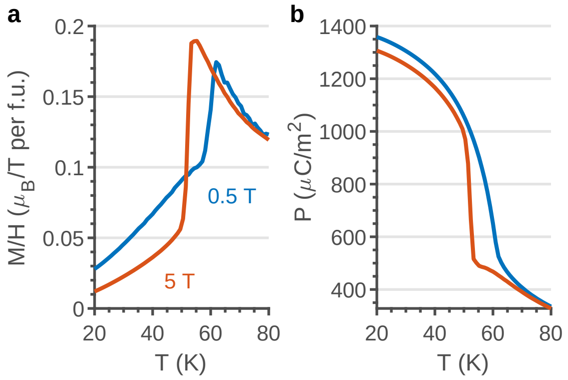

Supplementary figure 6: | **Temperature dependence of simulated magnetization and polarization of pure $\mathbf{Fe_2Mo_3O_8}$. a, b,** Temperature dependence of dc susceptibility M/H and polarization P, respectively. The calculations are performed for two different applied fields: 5 T (red) and 0.5 T (blue). The model parameters are $J_\perp = -1.2$ K and $J_\parallel = 47$ K.

Supplementary note 4

Polarization calculations:

We calculate the magnetically-induced electric polarization using the microscopic magnetoelectric coupling

$$H_{\rm me} = -g_{\parallel} P \sum_{\langle ij \rangle_{\parallel}} s_i \sigma_j - g_{\perp} P \sum_{\langle ij \rangle_{\perp}} s_i \sigma_j, \tag{S11}$$

where $\langle ij \rangle_{\parallel}$ and $\langle ij \rangle_{\perp}$ denote pairs of neighboring spins in the same ab layer and in neighboring layers, respectively, and the electric polarization P is aligned along the c-axis. The first term describes an additional polarization induced by the intralayer ordering present in both FiM and AFM phases (and even slightly above $T_{\rm N}$, see Fig. 1e of the manuscript), whereas the second term has different signs in the AFM and FiM states. The polarization is then given by,

$$P = P_0 + \chi_e \left(g_{\parallel} \sum_{\langle ij \rangle_{\parallel}} s_i \sigma_j + g_{\perp} \sum_{\langle ij \rangle_{\perp}} s_i \sigma_j \right), \tag{S12}$$

where P_0 is the electric polarization in the paramagnetic state and χ_e denotes the electric susceptibility.

The field dependence of the electric polarization calculated for $\chi_{\rm e} g_{\parallel} = -1000 \mu{\rm C/m^2}$ and $\chi_{\rm e} g_{\perp} = 400 \mu{\rm C/m^2}$ reproduces the experimentally observed polarization anomaly at the spin-flip transition in pure $Fe_2Mo_3O_8$ [2] (see Supplementary Fig. 5b). Supplementary Fig. 6b shows the temperature dependence of the polarization P in weak and strong magnetic fields. Note that the simulated polarization has opposite sign compared to Ref. [2] due to reversed crystal orientations.

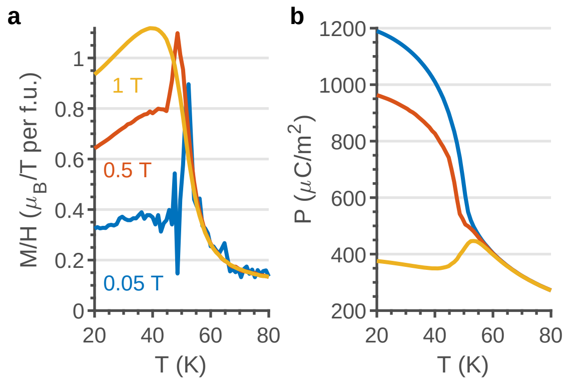

Supplementary figure 7: | **Temperature dependence of simulated magnetization and polarization of $Fe_{1.86}Zn_{0.14}Mo_3O_8$. a, b,** Temperature-dependent dc susceptibility M/H and polarization P for different magnetic fields, respectively. The model parameters for Zn concentration $x = 0.14$ are $J_{\perp} = -0.2$ K and $J_{\parallel} = 47$ K.

Supplementary Fig. 7b shows temperature dependence of the electric polarization for $Fe_{1.86}Zn_{0.14}Mo_3O_8$ calculated using $\chi_e g_{||} = -975\mu C/m^2$ and $\chi_e g_{\perp} = 525\mu C/m^2$.

[1] Wannier, G. H. The Statistical Problem in Cooperative Phenomena. *Rev. Mod. Phys.* **17**, 50–60 (1945).

[2] Kurumaji, T., Ishiwata, S. & Tokura, Y. Doping-tunable ferrimagnetic phase with large linear magnetoelectric effect in a polar magnet $Fe_2Mo_3O_8$. *Phys. Rev. X* **5**, 031034 (2015).
